# A hybrid organic-inorganic perovskite dataset

**DOI:** 10.1038/sdata.2017.57

**Published:** 2017-05-09

**Authors:** Chiho Kim, Tran Doan Huan, Sridevi Krishnan, Rampi Ramprasad

**Affiliations:** 1 Institute of Materials Science, University of Connecticut, 97 North Eagleville Rd., Unit 3136, Storrs, Connecticut 06269, USA

**Keywords:** Electronic devices, Atomistic models, Electronic structure, Density functional theory

## Abstract

Hybrid organic-inorganic perovskites (HOIPs) have been attracting a great deal of attention due to their versatility of electronic properties and fabrication methods. We prepare a dataset of 1,346 HOIPs, which features 16 organic cations, 3 group-IV cations and 4 halide anions. Using a combination of an atomic structure search method and density functional theory calculations, the optimized structures, the bandgap, the dielectric constant, and the relative energies of the HOIPs are uniformly prepared and validated by comparing with relevant experimental and/or theoretical data. We make the dataset available at Dryad Digital Repository, NoMaD Repository, and Khazana Repository (http://khazana.uconn.edu/), hoping that it could be useful for future data-mining efforts that can explore possible structure-property relationships and phenomenological models. Progressive extension of the dataset is expected as new organic cations become appropriate within the HOIP framework, and as additional properties are calculated for the new compounds found.

## Background and Summary

Perovskites belong to a class of inorganic crystals with chemical formula ABX_3_, sharing the same structure with calcium titanate CaTiO_3_. In such a perovskite structure, the inorganic cations A and B are coordinated by 12 and 6 anions X, respectively. By substituting an organic cation for A, the first hybrid organic-inorganic perovskites (HOIPs), namely CH_3_NH_3_PbX_3_ (X=Cl, Br, I), were synthesized and characterized in 1978 (ref. 1). HOIPs remained largely unnoticed until the first successful application of CH_3_NH_3_PbX_3_ (X=Cl, Br) as photovoltaic absorbers with a power conversion efficiency of 3.8% in 2009 (ref. 2). An enormous number of experimental and computational efforts have then been devoted to optimizing some halide-based HOIPs, e.g., CH_3_NH_3_PbI_3_, HC(NH_2_)_2_PbI_3_, and CH_3_NH_3_SnI_3_, for photovoltaic applications^3–6^. Currently, CH_3_NH_3_PbI_3_ and HC(NH_2_)_2_PbI_3_ have taken a leading position in providing high performance (reaching 20.1% in the conversion efficiency)^7^ and low fabrication cost^3–6^.

In fact, there are plenty of choices for the sites A, B, and X in a HOIP. At the site A, methylammonium CH_3_NH_3_^3–5,8^, formamidnium HC(NH_2_)_2_^7,9^, and many more^6^, have been realized. Cations B can be Pb or Sn while the halogens Br, I, and Cl can be used for X^1,2^. Moreover, the introduction of an organic cation A into the perovskite structure can give raise of many different structural motifs^6,10–12^, making the class of halide-based HOIPs highly diverse. Rapidly and thoroughly screening this un-explored domain of the chemical space, for instance, with the emerging data-driven approaches^13–25^, may reveal new promising compounds potentially meeting the pressing need for lead-free perovskite solar cell materials^26^.

This contribution aims at taking an initial step towards the creation of a comprehensive database of HOIPs, which may be useful for this goal. In fact, this idea has recently been emerging with some datasets of hybrid organic/inorganic perovskites, prepared at some level of computations^27,28^. Our dataset, which contains 1,346 HOIPs, is prepared uniformly at the level of density functional theory (DFT)^29,30^ from the initial structures predicted by the minima-hoping method^31,32^. For each material, the equilibrium structure, the relative energies ($\varepsilon_{{\mathrm{rel}}_{1}}$ and $\varepsilon_{{\mathrm{rel}}_{2}}$, computed with respect to different energy references as described in **Numerical calculations** Section), the atomization energy $\left(\varepsilon_{\mathrm{at}}\right)$, the dielectric constant (ε), and the direct or indirect energy bandgap (*E*_g_) are reported. This dataset, which is available at Dryad Digital Repository, NoMaD Repository, and Khazana Repository, can readily be expanded in multiple ways, i.e., new properties can be calculated from the provided structures, and new HOIPs can also be progressively added. We expect that this dataset can supply a playground for future machine learning based work in this active research area.

## Methods

### Workflow

**Figure 1:**
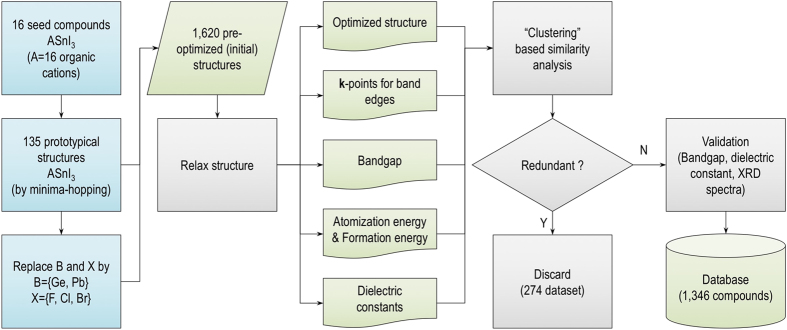
Scheme for preparing the dataset of hybrid organic-inorganic perovskites. Minima-hopping is a structure prediction method that was used for generating an initial set of 135 ASnI_3_ prototypical structures (where A stands for 16 organic cations), which were used as seeds for the creation of the remaining compounds.

**Figure 2:**
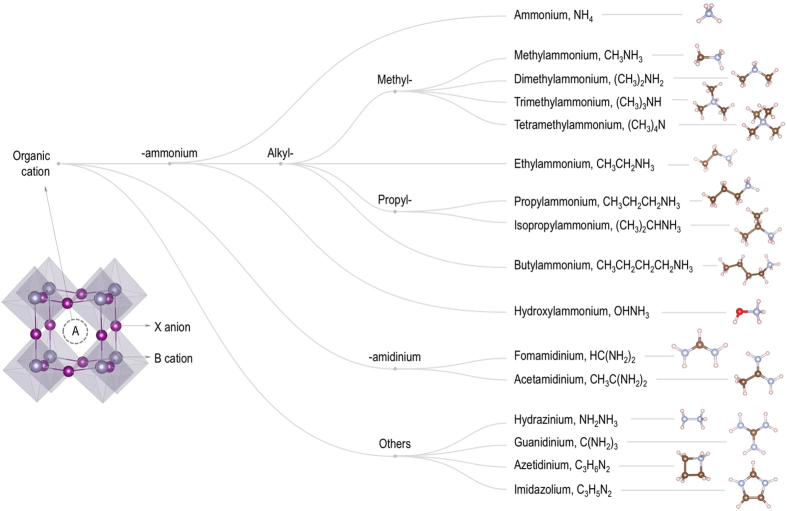
Ball and stick representations of 16 organic cations considered in the HOIP dataset. Carbon, hydrogen, oxygen and nitrogen atoms are shown in dark brown, light pink, red, and gray, respectively.

Figure 1 summarizes the workflow of the dataset preparation. This procedure starts by collecting 16 organic (molecular) cations A^+1^, all of which have been considered in the literature^1,6,7,12^. Each of these 16 cations, shown in Fig. 2, is placed at the site A of the ASnI_3_-based perovskites. This is the starting point for various structure prediction simulations, performed with the minima-hopping method^31,32^. The low-energy structures predicted for ASnI_3_ are subjected to a preliminary filtering step, keeping 135 prototype structures that are different in the DFT energy and the volume (these quantities are estimated on a not-so-high accuracy level used for the searches). Next, we expand the set of 135 structures by substituting either Ge or Pb for Sn, and, similarly, by substituting either F, Cl, or Br for I. The resulted 1,620 (initial) structures were optimized by DFT at the desired level of accuracy (described in **Numerical calculations** Section), yielding the relative energies and the atomization energies. Then, the band edge positions in the **k** space, the energy bandgap, and the dielectric constant were calculated for the optimized structures. A post-filtering step is finally performed on the whole dataset, removing redundancy (this time, redundancy is identified at the desired accuracy level of DFT computations), keeping 1,346 distinct data points (summarized in Table 1). Whenever possible, our calculated results are compared with those computed and/or measured data. Relaxed structures of all the materials are finally converted into the crystallographic information format (cif) using the pymatgen library^33^.

### Initial structure accumulation

As briefly demonstrated in the **Workflow** section, our dataset is built up from 135 prototype structures obtained by searching for low-energy structures of 16 HOIPs with chemical formulae ASnI_3_ (in fact, prototype structures of any material can be searched). In the minima-hopping structure prediction simulations, the DFT-level evergy is used to construct the potential energy surface (PES) of the composition^31,32^. Starting from an initial structure, low-energy minima of the PES are then searched by alternatively performing DFT-based local optimization runs (to locate the nearby minima) and molecular dynamics runs (to escape the identified minima). Thanks to some feedback mechanisms implemented, structure searches using this method is biased, giving some preference to the low-energy domains of the PES. Because of the large number of minima, the searches were performed at a given not-so-high accuracy level of DFT energy, and the minima identified in this step were then refined at the desired level. The power of the minima-hopping has been demonstrated over several classes of crystalline solids^34–36^, including three SnI_3_-based HOIPs^12^.

**Figure 3:**
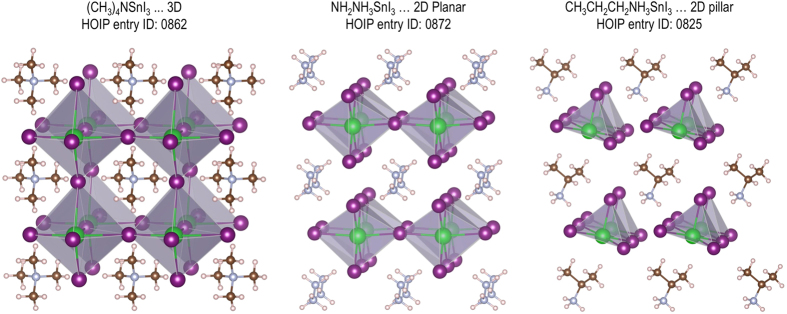
Lowest energy structures of tetramethylammonium, hydrazinium, and propylammonium tin iodide showing three prototypical conformations of organic-inorganic hybrid perovskites. Carbon, hydrogen, nitrogen, tin and iodine atoms are shown in dark brown, light pink, gray, green and purple, respectively.

For each of 16 ASnI_3_ HOIPs, numerous low-energy structures identified are subjected to a filtering step, keeping only those that are different by at least 5 meV/atom in the DFT energy and at least 0.1 Å^3^/atom in the structure volume. After the filtering step, 135 prototypical structures of 16 HOIPs were selected, three of which are shown in Fig. 3. In case of isotropic organic cations such as tetramethylammonium, a cubic-like cage formed by the network of Sn and I ions is stabilized in a three-dimensional structure. For the case of anisotropic or polar organic cations, the framework deforms into the two-dimensional planar or pillar motif. More structural variation is possible to be found from further structure searching using different organic cations and/or slightly nonstoichiometric composition in the HOIP system^6,37^. By substituting either Ge or Pb for Sn, and substituting either Cl, F, or Br for I, 1,620 structures of 192 *chemically distinct* HOIPs were obtained. They are the initial structures used to build up the HOIP dataset.

### Numerical calculations

#### General scheme

Our calculations are performed within the DFT^29,30^ formalism, using the projector augmented-wave (PAW) method^38^ as implemented in the Vienna *Ab initio* Simulation Package (vasp)^39–42^. The default accuracy level of our calculations is ‘Accurate’, specified by setting PREC=Accurate in all the runs with vasp. The basis set includes plane waves with kinetic energies up to 400 eV, as recommended by vasp manual for this level of accuracy. PAW datasets of version 5.2, which were used to describe the ion-electron interactions, are also summarized in Table 2. The van der Waals dispersion interactions are estimated with the non-local density functional vdW-DF2 (ref. 43). The generalized gradient approximation (GGA) functional associated with vdW-DF2, i.e., refitted Perdew-Wang 86 (rPW86)^44^, was used for the exchange-correlation (XC) energies. For all the calculations, except bandgap determination, we sample the Brillouin zones, which are significantly different in shape for the different compounds, by an equispaced (with the spacing of *h*_k_=0.20 Å^−1^), Γ-centered Monkhorst-Pack^45^ **k**-points mesh. The equilibration of the examined structures is assumed when the atomic forces are below 0.01 eV/Å. This numerical scheme is consistent with that we used for preparing the polymer dataset reported in ref. 35.

#### Bandgap determination

The bandgap *E*_g_ is perhaps the most desired physical property of HOIPs. Within DFT, *E*_g_ is determined as the energy difference between the conduction band minimum (CBM) and the valence band maximum (VBM), identified on a given **k**-point mesh. For a solid with an arbitrary primitive cell, the locations of VBM and CBM are generally not known beforehand, and the **k**-point mesh should be very dense in order to locate the band edges accurately. With a mesh of this type, the computation of *E*_g_ using the Heyd-Scuseria-Ernzerhof (HSE06)^46,47^ exchange-correlation functional, the level of DFT at which the calculated bandgap is expected to be close to the real bandgap, is computationally prohibitive. Although such a computation at the GGA level of DFT is feasible, *E*_g_ is generally underestimated by 30% or more^48^.

**Figure 4:**
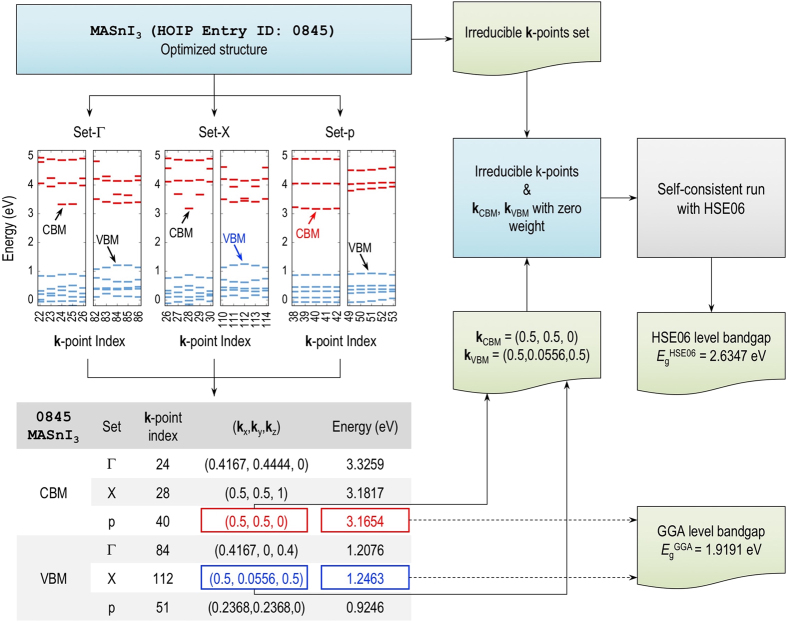
Scheme for calculation of the bandgap of hybrid organic-inorganic perovskites at GGA and HSE06 level of theories. Data entry 0,845 (MASnI_3_; CH_3_NH_3_SnI_3_, Khazana ID: 2,695) is used for demonstration. Set-Γ, Set-X and Set-p correspond to the **k**-points sets generated within Γ-centered mesh, X-centered mesh, and high symmetry path for *P*_1_ group.

The conduction bands and the valence bands computed at the GGA and HSE06 levels of DFT are essentially similar in the shape. However, they are shifted as a whole with respect to each other and to the true electronic structrures (see, for example, ref. 49). Therefore, our bandgap determination procedure, shown in Fig. 4, includes two steps. First, the locations of VBM and CBM are searched at the GGA level on three different dense **k**-point meshes. The first two meshes (one centered at Γ=(0,0,0) and the other centered at X=(0.5, 0.5, 0.5)) are equispaced with *h*_k_=0.15 Å^−1^, while the third mesh contains **k**-points distributed along Γ-X-M-Γ-R-M-X-R, the path that has widely been used to represent the electronic band structrure of HOIPs^12,50^. In the second step, the positions of VBM and CBM identified in the first step are used with zero weight for sampling the Brillouin zones using a Monkhorst-Pack **k**-point mesh with *h*_k_=0.20 Å^−1^, hereby determining the energy difference between CBM and VBM at the HSE06 level of DFT. Although this procedure needs some extra work, we expect that the bandgap computed for HOIPs with an arbitrary primitive cell is reliable.

#### Atomization and relative energies definitions

The atomization energy of each of these compounds are calculated as

(1)εat=EABX3−∑iniEi

where $E_{{\mathrm{ABX}}_{3}}$ is the energy of the HOIP and *n*_*i*_ and *E*_*i*_ are the number and the energy of an isolated atom of the element *i* respectively. We also report two kinds of relative energies with respect to the atomic constituents and solid constituents.

(2)εrel2=EABX3−EA′−EB−32EX2−12EH2

(3)εrel2=EABX3−EA′−EBX2−EHX

where $E_{A\prime }$, *E*_B_, $E_{X_{2}}$, and $E_{H_{2}}$ are the energies of isolated neutral organic molecule A, metallic crystals B, isolated X_2_, and H_2_ molecules respectively. $E_{{\mathrm{BX}}_{2}}$ and *E*_HX_ are the energies of the metallic halides (BX_2_) and hydrogen halides (HX), respectively. For the case of tetramethylammonium cation (C_4_H_12_N^+^), the energy of neutral trimethylamine (C_3_H_9_N) was used for $E_{A\prime }$, and the energies of the molecules C_2_H_6_ and CH_3_X are used instead of $E_{H_{2}}$ and *E*_HX_ in equations (2) and (3), respectively.

### Post-filtering

The preliminary filtering step is performed only on prototypical structures (ASnI_3_) based on their DFT energy and bandgap estimated during the structure prediction runs with a limited accuracy. Therefore, an additional filtering step is performed on the whole relaxed structures from 1,620 initial structures to remove any possible redundancy. Within this step, all cases with the same chemical composition but different by less than 2% in volume of unit cell Ω, *E*_g_, $\varepsilon_{\mathrm{at}}$, ${є}_{\mathrm{elec}}$ and ${є}_{\mathrm{ion}}$, are clustered. All the clustered points were inspected visually, keeping only those materials that are distinct. At the end of this step, we are left with 1,346 distinct compounds (also summarized in Table 1). These compounds constitute our final dataset.

## Data Records

The complete dataset of HOIP materials can be downloaded as a tarball or can be accessed via Dryad Digital Repository (Data Citation 1) and Khazana Repository (http://khazana.uconn.edu/). 1,346 compounds in our final dataset are recorded in Khazana ID from 1,851 to 3,197. All 8,076 (=1,346×6) DFT runs of the whole dataset (for each structure, there are 6 runs, including relax, dielectric, GGA bandgap with Γ-centered mesh, GGA bandgap with X-centered mesh, GGA bandgap with **k**-points distributed along Γ-X-M-Γ-R-M-X-R, and HSE06 bandgap) are hosted by NoMaD Repository (Data Citation 2).

### File format

The information reported in the dataset for a given material is stored in a file, named as N.cif, where N is a cardinal number used for the identification of the entry in the dataset. The first part of a file of this type is devoted to the optimized structure in the standard cif format which is compatible with many visualization software. Other information, including the calculated properties, is provided as the comments lines in the second part of the file as follows (for the example of N=845).

While most of the keywords are clear, we used keyword Label to provide more detail information of the HOIP compounds, which includes the common name of A organic cation, B cation and X anion. The origin of the formula and structure of organic cations is provided in the keyword Organic cation source. Keywords Material class and Geometry class are set to be ‘Hybrid organic-inorganic perovskite’ and ‘Bulk crystalline materials’, respectively.


# HOIP entry ID:                      0845
# Khazana ID:                         2695
# Organic cation source:              T.D.Huan et al., Phys. Rev. B 93,094105(2016)
# Label:                              Methylammonium Tin Iodide
# Material class:                     Hybrid organic-inorganic perovskite (MC_ino)
# Geometry class:                     Bulk crystalline materials (GC_cry)
# Organic cation chemical formula:    CH3NH3
# Number of atom types:               5
# Total number of atoms:              12
# Atom types:                         C H N Sn I
# Number of each atom:                1 6 1 1 3
# Bandgap, HSE06 (eV):                2.6347
# Bandgap, GGA (eV):                  1.9191
# Kpoint for VBM:                     0.5, 0.0556, 0.5
# Kpoint for CBM:                     0.5, 0.5, 0
# Dielectric constant, electronic:    4.8562
# Dielectric constant, ionic:         13.0716
# Dielectric constant, total:         17.9278
# Refractive index:                   2.2037
# Atomization energy (eV/atom):       −3.9099
# Relative energy1 (eV/atom):         0.2785
# Relative energy2 (eV/atom):         0.4387
# Volume of the unit cell (A^3):      251.45
# Density (g/cm^3):                   3.51.


### Graphical summary of the dataset

**Figure 5:**
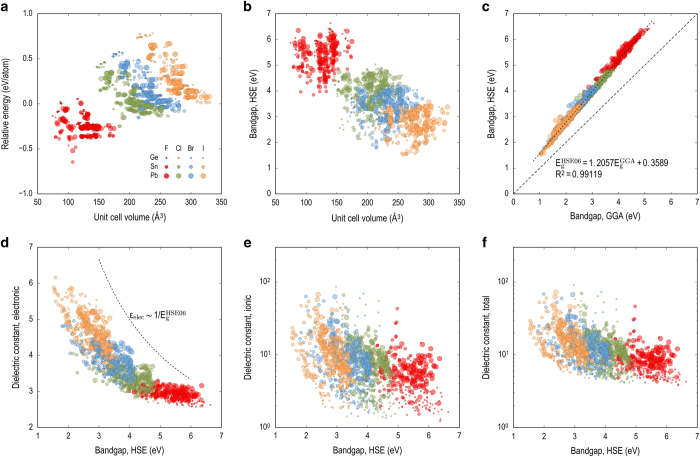
A summary of the HOIP dataset based on the calculated volume of unit cell Ω, relative energy $\varepsilon_{{\mathrm{rel}}_{1}}$, **GGA level bandgap** $E_{g}^{\mathrm{GGA}}$**, HSE level bandgap** $E_{g}^{\mathrm{HSE06}}$**, and the dielectric constants** ${є}_{\mathrm{elec}}$, ${є}_{\mathrm{ion}}$, **and** $є={є}_{\mathrm{elec}}+{є}_{\mathrm{ion}}$. The panels show (**a**) unit cell volume vs relative energy, (**b**) unit cell volume vs HSE bandgap, (**c**) GGA bandgap vs HSE bandgap, (**d**) HSE bandgap vs electronic dielectric constant, (**e**) HSE bandgap vs ionic dielectric constant, and (**f**) HSE bandgap vs total dielectric constant. In each plot, the color and size of the symbols are coded following the figure keys shown in plot (**a**).

We visualize the calculated quantities in the property space as shown in Fig. 5. Because the relative energy, unit cell volume of the compound, bandgap and dielectric constant are the primary properties reported by this dataset, six plots, namely $\Omega -\varepsilon_{{\mathrm{rel}}_{1}}$, Ω−EΕgHSE06, $E_{g}^{\mathrm{GGA}}-E_{g}^{\mathrm{HSE06}}$, $E_{g}^{\mathrm{HSE06}}-{є}_{\mathrm{elec}}$, $E_{g}^{\mathrm{HSE06}}-{є}_{\mathrm{ion}}$, and $E_{g}^{\mathrm{HSE06}}-є$, were shown. Compounds containing different A cations and X anions are represented using different colors and size of the symbols to clarify the role of the chemical contents in controlling the properties of the HOIP.

It can be clearly seen that the dataset is clustered based on the X anions, showing the sequence of F, Cl, Br and I. As shown in Fig. 5a most of F containing HOIP compounds are more favorable to be formed as measured by the relative energy regardless of the A cation contents. Bandgap and unit cell volume are strongly correlated mainly because the electronegativity and the ionic radii of X anions significantly differ for F, Cl, Br and I. Simple and strong correlation between GGA and HSE level bandgap is found as a linear function with scale factor of ~1.2 as shown in Fig. 5c. Small bandgap values varying from 1.5 eV to 1.6 eV, favorable for photovoltaic application, was found for SnI_3_ containing HOIP compounds including CH_3_NH_3_SnI_3_, NH_3_NH_2_I_3_SnI_3_, C_3_H_8_NSnI_3_. A limit of the form $є\mathrm{elec}\sim 1/E_{g}^{\mathrm{HSE06}}$ shown in Fig. 5(d) has also been demonstrated for other classes of materials in the literature^13,35,36,51–62^.

## Technical Validation

**Figure 6:**
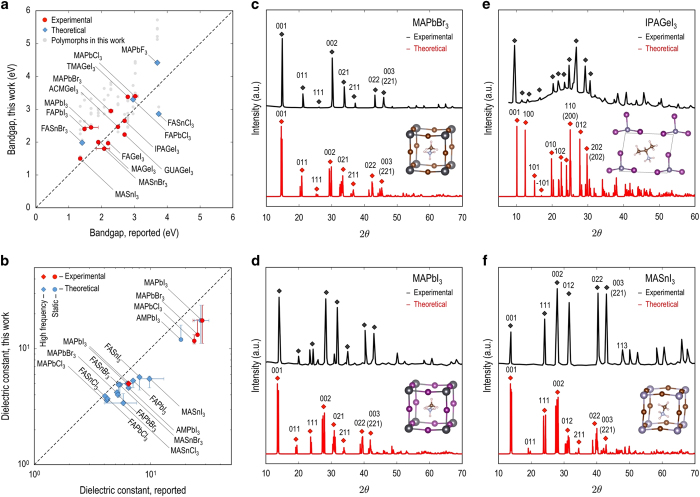
Validation of data computed for some HOIPs by comparing it with the measured data available. Bandgap and dielectric constants computed for the low-energy structures of these compounds are plotted in (**a**,**b**) vs. those experimentally measured, respectively. In these panels, the lowest-energy structure of each HOIP is indicated by a colored symbol while data from the energetically competing structures are shown in gray (**a**) or given within an error bar (**b**). Experimental data of bandgap and dielectric constants of these HOIPs is obtained from refs 8,64,65,66,67 68,69 70,71 72,73 74,75 76,77 78,79 and refs 74–83, respectively. In (**c**–**f**), the simulated and measured XRD spectra for MAPbBr_3_^65^, MAPbI_3_^84^, IPAGeI_3_^73^, and MASnI_3_^5,85^, are shown. The reported index of reflection orientation is given on top of each significant peak.

The relative energy computed via equation (2) is physically relevant to examine the relative stability useful for future studies of new HOIPs. As the dataset contains theoretically stable structures, we used the bandgap, dielectric constant, and XRD pattern with Cu K*α* (1.54056 Å) for the validation of the calculations. Since available experimental studies for HOIPs seem to be limited to a small subset of the combinatorial possibilities, a small number of experimental bandgap could only be collected from available resources. These correspond to compounds containing acetamidinium (ACM, C_2_H_7_N_2_), formamidinium (FA, CH_5_N_2_), guanidinium (GUA, CH_6_N_3_), isopropylammonium (IPA, C_3_H_10_N), methylammonium (MA, CH_3_NH_3_), and tetramethylammonium (TMA, C_4_H_12_N). Four computed bandgaps are also included in the comparison set. As shown in Fig. 6a, the calculated bandgap for the most stable structure of each case (marked as color coded symbols) agrees well with the data from previous studies. (gray symbols correspond to less stable polymorphs).

In order to further validate the HOIP dataset, experimentally measured and theoretically calculated dielectric constants for both high frequency and static regime are collected and compared with computed dielectric constants. The information is available for a limited number of HOIPs with MA and FA organic cations. Since the computation of dielectric constant using DFPT is highly sensitive to the numerical accuracy of the vibration frequency we used rather tight convergence criterion for the change of total energy by 10^−8^ eV. Figure 6b shows the excellent agreement between previously reported and computed dielectric constants for the selected HOIPs. Finally, we show the XRD spectra calculated for four HOIPs, including MAPbBr_3_, MAPbI_3_, IPAGeI_3_ and MASnI_3_ in Fig. 6c–f. Each of them is compared with the corresponding measured XRD patterns showing comparable agreement that can be regarded as supportive validation of computational schemes.

## Usage Notes

This dataset, which includes 1,346 HOIPs, has been consistently prepared using first-principles calculations. While the HSE06 bandgap $E_{g}^{\mathrm{HSE06}}$ is believed to be fairly close to the true bandgap of the materials, the GGA-rPW86 bandgap is also reported for completeness and for further possible analysis. The reported atomization energy and the dielectric constants are also expected to be accurate.

## Additional Information

**How to cite this article:** Kim, C. *et al.* A hybrid organic-inorganic perovskite dataset. *Sci. Data* 4:170057 doi: 10.1038/sdata.2017.57 (2017).

**Publisher’s note:** Springer Nature remains neutral with regard to jurisdictional claims in published maps and institutional affiliations.

## Supplementary Material



## Figures and Tables

**Table 1:** Summary of the data subclasses in the hybrid organic-inorganic perovskites dataset.

**Organic cation A**	**Cation B and anion X**				**Total**								
	**Ge**					**Sn**				**Pb**			
	**F**	**Cl**	**Br**	**I**		**F**	**Cl**	**Br**	**I**	**F**	**Cl**	**Br**	**I**
Ammonium	2	2	4	3	3	4	3	4	2	2	3	3	35
Methylammonium	6	5	6	6	6	6	6	6	6	5	5	6	69
Dimethylammonium	5	7	9	8	9	8	7	8	5	7	7	7	87
Trimethylammonium	7	8	9	9	7	10	11	11	9	11	9	12	113
Tetramethylammonium	2	3	3	2	1	3	3	2	1	3	3	3	29
Ethylammonium	9	10	11	12	11	11	12	12	12	10	10	11	131
Propylammonium	8	11	13	12	10	13	13	12	11	10	11	13	137
Isopropylammonium	9	8	9	10	9	9	11	9	12	11	8	10	115
Butylammonium	4	3	3	4	4	3	3	3	2	4	4	4	41
Hydroxylammonium	7	7	7	7	5	6	7	7	7	6	7	7	80
Formamidinium	2	3	3	3	2	3	3	3	2	3	3	3	33
Acetamidinium	6	5	6	6	6	6	7	6	5	6	6	6	71
Hydrazinium	8	10	11	11	8	11	12	11	8	9	8	9	116
Guanidinium	3	3	3	3	2	2	3	3	2	3	3	3	33
Azetidinium	13	14	16	15	14	17	16	16	9	13	13	15	171
Imidazolium	6	6	8	8	9	8	7	9	6	7	5	6	85
Total	97	105	121	119	106	120	124	122	99	110	105	118	1,346

**Table 2:** VASP PAW potentials of the elements used for calculations in this work.

**Element**	**POTCAR**	**Element**	**POTCAR**	**Element**	**POTCAR**
Bromine	Br	Carbon	C	Chlorine	Cl
Fluorine	F	Germanium	Ge_d	Hydrogen	H
Iodine	I	Nitrogen	N	Oxygen	O
Lead	Pb_d	Tin	Sn_d		
